# Repeated Home-Applied Dual-Light Antibacterial Photodynamic Therapy Can Reduce Plaque Burden, Inflammation, and aMMP-8 in Peri-Implant Disease—A Pilot Study

**DOI:** 10.3390/cimb44030085

**Published:** 2022-03-08

**Authors:** Hanna Lähteenmäki, Tommi Pätilä, Ismo T. Räisänen, Esko Kankuri, Taina Tervahartiala, Timo Sorsa

**Affiliations:** 1 Department of Oral and Maxillofacial Diseases, University of Helsinki and Helsinki University Hospital, 00280 Helsinki, Finland; ismo.raisanen@helsinki.fi (I.T.R.); taina.tervahartiala@helsinki.fi (T.T.); timo.sorsa@helsinki.fi (T.S.); 2 Department of Pediatric Heart Surgery and Organ Transplantation, New Children’s Hospital, Helsinki University, 00100 Helsinki, Finland; tommi.patila@helsinki.fi; 3 Department of Pharmacology, Helsinki University, 00100 Helsinki, Finland; esko.kankuri@helsinki.fi; 4 Division of Periodontology, Department of Dental Medicine, Karolinska Institutet, 141 52 Huddinge, Sweden

**Keywords:** antibacterial photodynamic therapy, aMMP-8, oral hygiene, peri-implant disease

## Abstract

Until now, in clinical dentistry, antibacterial photodynamic therapy (aPDT) has been restricted to in-office treatments, which hampers repeated applications. This pilot study tested the benefit of a commercially available Lumoral^®^ device designed for regular periodontal dual-light aPDT treatment at home. Seven patients with peri-implant disease applied dual-light aPDT daily in addition to their normal dental hygiene for four weeks. A single Lumoral^®^ treatment includes an indocyanine green mouth rinse followed by 40 J/cm^2^ radiant exposure to a combination of 810 nm and 405 nm light. A point-of-care analysis of active-matrix metalloproteinase (aMMP-8), visible plaque index (VPI), bleeding on probing (BOP), and peri-implant pocket depth (PPD) measurements was performed on day 0, day 15, and day 30. Reductions in aMMP-8 (*p* = 0.047), VPI (*p* = 0.03), and BOP (*p* = 0.03) were observed, and PPD was measured as being 1 mm lower in the implant (*p* = ns). These results suggest a benefit of regular application of dual-light aPDT in peri-implantitis. Frequently repeated application can be a promising approach to diminishing the microbial burden and to lowering the tissue destructive proteolytic and inflammatory load around dental implants. Further studies in larger populations are warranted to show the long-term benefits.

## 1. Introduction

Dental implants have been used to replace lost teeth for decades [1]. Dental implants’ durability is excellent, and their success rate is high. Still, inflammation at the peri-implant tissues is frequent [2,3]. Dental implant health is assessed similar to periodontal tissues around natural teeth: by measuring the peri-implant probing pocket depth (PPD), bleeding on probing (BOP), suppuration, and radiographic findings [4,5]. The primary etiologic reason for the progression of bone destruction and the inflammation of peri-implant tissue is the accumulation of oral biofilm. Peri-implant disease results from bacterial infection and develops due to the host immune response to an overwhelming bacterial insult. The elimination of bacterial biofilm from the implant surface is the primary objective when treating peri-implant disease [6,7].

Antibacterial photodynamic therapy (aPDT) and antibacterial blue light (aBL) are emerging treatment methods auxiliary to mechanical debridement for periodontitis and peri-implantitis [8,9,10,11,12,13]. The aPDT is a combination of light and externally applied light photosensitizer. Indocyanine green (ICG) is a widely used aPDT photosensitizer in dentistry due to low toxicity, water solubility, and light absorption at the near-infrared (NIR) wavelengths, which are safe and have good tissue penetration [10,14]. The light energy absorbed by ICG is discharged either as a thermal vibration or by further moving to the outer-most electron ring of a nearby oxygen molecule, thus mediating the antibacterial effects [8,9,10,11,12,14,15,16]. The method of action in aBL is based on the absorption of light by bacterial cells [17]. The photosensitizing molecules within the bacteria are considered to be primarily porphyrins and flavins. The combined use of aBL and aPDT has shown an enhanced antibacterial action with the ability to provide a sustained antibacterial effect on a bacterial biofilm [16,18,19].

Collagenase-2/neutrophil collagenase or matrix metalloproteinase (MMP)-8 is a collagenolytic host proteinase. The active form (aMMP-8) reflects and predicts periodontal tissue destruction and inflammation. The aMMP-8 is a promising candidate biomarker used to diagnose, grade, and predict periodontitis and peri-implantitis as well as to monitor their treatments [20,21,22,23,24,25,26,27,28,29]. Chairside aMMP-8 test kits (PerioSafe^®^/ImplantSafe^®^/ORALyzer^®^) are commercially available as quantitative point-of-care kits for periodontitis and dental peri-implantitis. Accurate quantitative reading of aMMP-8 can be conveniently performed chairside [20,28,30,31]. An increased level of aMMP-8 is an initial sign of an inflammatory reaction in the periodontium. Several studies support the diagnostic validity of aMMP-8 peri-implant sulcular fluid analyses in diagnosing peri-implant disease and in differentiating healthy and diseased dental implant patients [20,21,22,23,24,25,26,27,28,29,32].

Repeated antibacterial treatment by dual-light aPDT performed at home can be used to improve oral hygiene. The aim of the study was to preliminarily test the ability of the anti-plaque and anti-inflammatory effects of the daily application of dual-light aPDT in peri-implant disease patients, monitored by the aMMP-8 point-of-care method. We hypothesized that improved oral hygiene performance by dual-light aPDT would reduce collagenolytic inflammation and burden without adverse effects.

## 2. Materials and Methods

The study was conducted in accordance with the ethical principles of the Declaration of Helsinki. The study was approved by the ethics committee of the Hospital District of Helsinki and Uusimaa (HUS/1271/2019), and all participants provided written informed consent before enrolment.

### 2.1. Study Design and Participants

This experimental pilot study followed an interventional pre-post protocol. The inclusion criteria were (1) implant patients arriving for a maintenance visit at a dental clinic (Hammasklinikka Kruunu, Tampere, Finland); (2) a diagnosis of peri-implant mucositis or peri-implantitis by a dentist according to the definitions described below; and (3) written informed consent. The exclusion criteria included (1) smoking; (2) diabetes; (3) a need for a hopeless teeth extraction, or open cavities in need of immediate endodontic treatment; or (4) the presence of a major physical limitation or restriction that prohibits the hygiene procedures used in the study protocol. Seven patients were enrolled, four males and three females. Their ages ranged from 65 to 89 years. A single implant with screw crown in each patient was selected for the study, with three implants being from the mandibulae and five being from the maxilla. All implants were produced by Nobel Biocare^®^. The patients were asked to continue with their regular home care and routine oral hygiene habits during the study period. The patients were instructed to perform an improved oral hygiene protocol by including dual-light aPDT in their regular home care for four weeks before the actual mechanical maintenance treatment. The patient characteristics are presented in Table 1.

### 2.2. Dental Examination

The dental examinations were performed at the beginning of the study (time point 1), after 15 days (time point 2), and after 30 days (time point 3). An oral clinical investigation at each time point included an assessment of the visible plaque index (VPI) (scaling 0–3 on all tooth surfaces), BOP (yes/no, 1/0), and PPD (mm). A millimeter-grade Ball-Tip Screening probe, WHO, AEEP23/WHOBX (American Eagle Manufacturing Co., New Bern, NC, USA) was used. A digital intra-oral radiograph was acquired using Sirona Heliodent plus (Dentsply Sirona, New York, NY, USA), and Soredex Digora Optime (KaVo Dental GmbH, Biberach, Germany) software was used for the analysis of alveolar bone destruction to define peri-implantitis [33,34]. Clinical photographs were shot at all time points with an iPhone^®^ 8 mobile phone 12 MP camera with f/1.8 aperture, six-element lens, optical stabilization, and Wide Color capture deployment (Apple Inc., Cupertino, CA, USA).

The diagnostic definition of peri-implant health and disease was based on the criteria published by Renvert et al. [33] and Berghlundh et al. [34]. Healthy peri-implant tissue was defined by the absence of peri-implant signs of soft tissue inflammation, including redness, swelling, and profuse BOP, together with the absence of further additional bone loss following initial healing. The definition of peri-implant mucositis (*n* = 3) was based on the presence of peri-implant signs of inflammation described above in combination with a lack of additional bone loss following initial healing. Peri-implantitis (*n* = 4) was defined by the presence of peri-implant signs of inflammation with radiographic evidence of bone loss (>2 mm) and increased probing depths ≥ 3 mm with the presence of BOP, swelling, and possible suppuration [33,34].

### 2.3. Improved Oral Hygiene Protocol by Dual-Light Photodynamic Treatment

**Figure 1:**
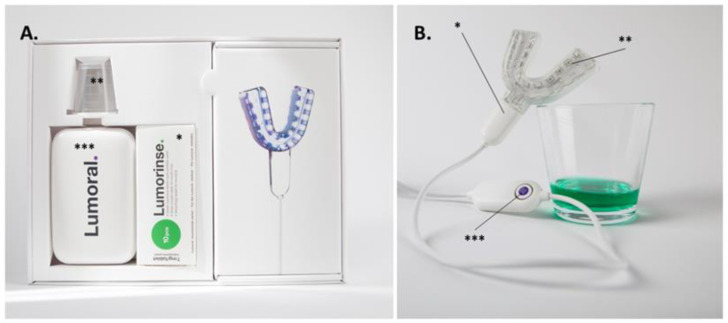
The dual-light aPDT device was used in the study. (**A**) The packaging provided for the study subjects included the effervescent ICG tablets (*) to be dissolved in 30 mL of water, for which a measuring cup (**) was provided. A power source (***) was provided for the mouthguard-type light applicator (**B**). The mouthpiece (*) is composed of 48 LED components (**) able to provide 405 nm and 810 nm light simultaneously. Symmetrically assembled LEDs provided light for both the maxillary and mandibular dental arches. The button on the control unit provided a treatment time of 10 min (***). Dissolved mouth rinse can be seen in the glass.

Antibacterial photodynamic therapy was performed using Lumorinse^®^ mouthwash (Lumoral, Espoo, Finland) in conjunction with a Lumoral^®^ light applicator (Lumoral, Espoo, Finland). Lumorinse^®^ (Lumoral, Espoo, Finland) is a CE-marked effervescent tablet including 7 mg of ICG and is used by dissolving in 30 mL of water, which produces a mouthwash with an ICG concentration of 250 µg/mL. The mouthwash is swished for 1 min, enabling ICG to adhere to dental plaque before light application. Lumoral^®^ (Lumoral, Espoo, Finland) is a CE-marked medical device providing simultaneous 405 nm aBL and 810 nm near-infrared (NIR) LED light in a mouthguard form. Together with Lumorinse^®^ (Lumoral, Espoo, Finland), the product offers simultaneous aBL and aPDT action on dental plaque. The Lumoral^®^ light applicator is placed in the mouth, and during 10 min use, the device delivers a radiant exposure of 40 J/cm^2^ light with approximately half of the light energy as aBL and half as NIR light. Thus, the device gives light to the whole dentition, with the 48 LEDs illuminating both dental arches (Figure 1).

In addition to their regular homecare habits, the study subjects were asked to use the dual-light aPDT method once a day for the first two weeks until time point 2, after which they used the method twice daily until time point 3. The device use was integrated into home care in such a way that the study subjects were asked to brush their teeth as usual after each use of the device. Subjects’ commitment to the treatment protocol and to the device use was checked by interview and was based on the subject’s statement.

### 2.4. Peri-Implant Sulcus Fluid (PISF) Collection and Analysis Samples

The ImplantSafe^®^ test (Dentognostics, Jena, Germany) and ORALyzer^®^ digital aMMP-8 reader (Dentognostics, Jena, Germany) were used to quantify aMMP-8 (ng/mL) in PISF. The PISF samples were obtained by inserting a collection strip into the peri-implant pocket in the sulcus point exhibiting the worst clinical situation for 30 s. Then, according to the manufacturer’s instructions, the strip was place in the elution fluid for five minutes. After five minutes of incubation, the elution tube was swirled a couple of times. The dipstick was then immersed into the elution fluid for 15 s and placed into the reading compartment of the ORALyzer^®^ device (Dentognostics, Jena, Germany). The ImplantSafe^®^ test (Dentognostics, Jena, Germany) can read qualitatively after 5 min; the reading window shows one blue line (test is valid, test negative, 20 ng/mL) or two lines (test is valid, test positive, ≥20 ng/mL). The ORALyzer^®^ device (Dentognostics, Jena, Germany) read the test quantitatively. The aMMP-8 enzyme test results were documented by photography (iPhone 8).

### 2.5. Statistical Methods

SAS 9.4 software was used for the power calculations (SAS Institute, Cary, NC, USA) and GraphPad software version 9.1.0 (GraphPad Software, La Jolla, CA, USA) to analyze the data and to create the graphs. A Wilcoxon nonparametric analysis of paired groups was performed to compare the difference of continuous variables (aMMP-8, VPI, and PPD) and chi-square contingency analysis for the dichotomous variable (BOP) between the pretreatment and final measurements. A *p*-value less than 0.05 was considered statistically significant.

We performed a power calculation for the required sample size based on our recently published study on ICG-assisted aPDT on dental plaque [35]. In this study, we evaluated the aMMP-8 levels four days after repeated aPDT administration. We acknowledged the difficulty of estimating the sample size based on somewhat different patient populations and treatment durations. Moreover, we considered that the current study was intended as a pilot for peri-implant disease and involved a longer duration of treatment and that the same patients acted as their own representative controls.

**Figure 2:**
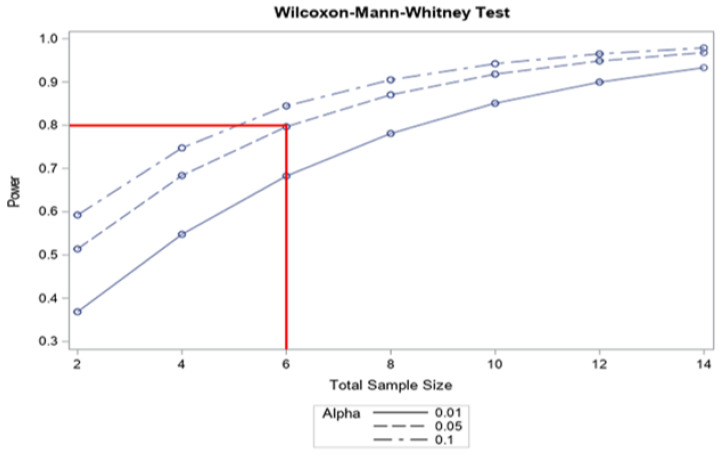
The power calculation.

Calculated (SAS 9.4, SAS Institute, Cary, NC, USA) from the previous study design and assigning 5% for alpha errors and 20% for TYPE II errors (80% power), we arrived at a sample size of six patients (Figure 2). To allow for possible dropout, we chose to recruit seven patients to this pilot study.

## 3. Results

All seven participants, aged 65–89 years, completed the whole protocol. None of the participants had diabetes or rheumatoid arthritis, and they were all non-smokers. One had asthma, and two had a diagnosis of heart disease. The year of placement of the prosthetic reconstruction ranged from 2009 to 2016, with a median of 2.62 years since placement.

At the beginning of the study (time point 1), all study subjects had gingival bleeding at the area of the selected implant (BOP 7/0). The amount of dental plaque by VPI ranged from 0 to 2, with a median of 1. The probing depth ranged from 2 to 6 mm with a median of 4 mm. The aMMP-8 ranged from 10 to 170.8 ng/mL, with a 72.6 ng/mL median. In four implants, X-ray imaging showed >2 mm peri-implant bone loss from the estimated bone level.

At time point 2, after two weeks of once-a-day dual-light aPDT use, two subjects showed negative BOP at the area of the selected implant (BOP 5/2). One patient showed a reduction in plaque, with VPI reducing from 1 to 0. The others had no change in the amount of dental plaque. The probing depth stayed the same in all subjects. The aMMP-8 level measurements ranged from 10 to 138.9 ng/mL, with a 37.1 ng/mL median.

**Figure 3:**
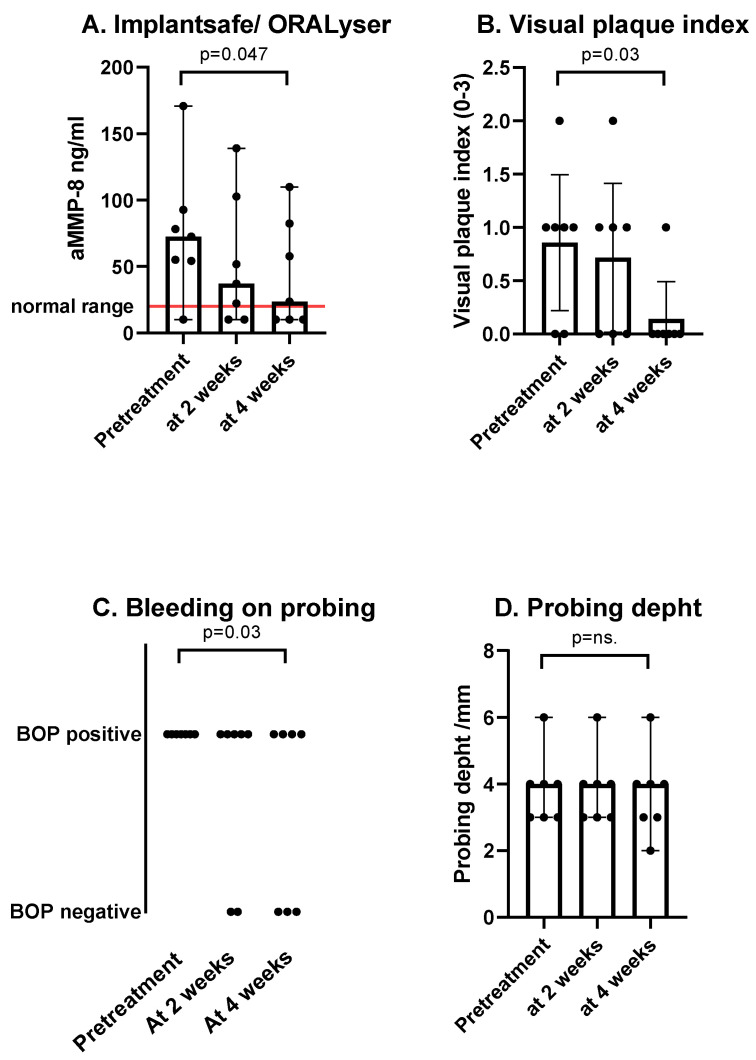
The dots represent the measured individual values, the bars represent the mean, and the error bars are the standard error of the mean. (**A**) There was a reduction in aMMP-8 measured from PISF between the pretreatment and after four weeks of treatment. The red line represents the normal value in PISF measurements in implant patients. (**B**) After two weeks of single-use of the dual-light aPDT device, a suggestive reduction in VPI was measured. After the subsequent use of twice a day, VPI was significantly lower. (**C**) Bleeding on probing was positive in all study subjects at the beginning of the study. However, it was measured negative in three patients at four weeks. (**D**) One implant pocket showed a reduction in PD during the four-week study period.

**Figure 4:**
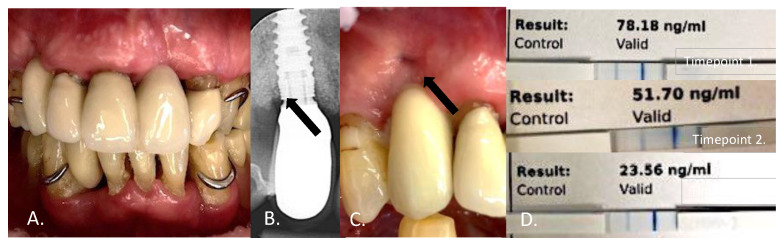
This 89-year-old patient (**A**) showed buccal bone loss, assessed using an intraoral X-ray (**B**), and a gingival fistula at the study implant D13 (**C**). The visual plaque index measured 1/3 at the beginning of the study, with a probing depth of 4 mm. The aMMP-8 value in the peri-implant sulcular fluid was 78.18 ng/mL at the beginning of the study **(D)**. After two weeks of Lumoral^®^ dual-light aPDT treatment repeated once a day, the aMMP-value was 51.70 (**D**), after which the Lumoral^®^ treatment was repeated twice a day for the next two weeks. At four weeks, the aMMP-8 was 23.76 ng/mL, and the visual plaque index measured 0/3. In this patient, bleeding on probing was measured positive though the whole study period, and no change was observed in the probing depth.

At time point 3, after another two weeks of regular dual-light aPDT use but now twice a day, three subjects had no bleeding at the gingiva around the studied implant (BOP 4/3). VPI showed a level-one plaque score in one patient, while all others had scores of 0. One patient had a reduction of 1 mm in probing depth, while others showed no change. The aMMP-8 level measurements ranged from 10 to 109.8 ng/mL, with a median of 23.6 ng/mL (Figure 3 and Figure 4). No adverse events were observed.

## 4. Discussion

This initial study suggests a benefit of home-applied dual-light aPDT in patients with peri-implant disease. We observed improved hygiene in all subjects measured by a reduction in VPI. The aMMP-8 in PISF levels were clearly reduced, being in line with the decrease in inflammation measured by BOP. In one implant, a 1 mm reduction in PPD was measured four weeks after the beginning of the study. The significance of this observed reduction in inflammation lies in none of the study subjects receiving any professional treatment until after the study. All improvements were achieved at home by enhanced oral hygiene.

The root cause of peri-implant disease is tissue reaction to bacterial biofilm. Mechanical biofilm removal is the standard of care for disease management and aims for a reduction in bacterial colonization on the surface of the implant [35,36]. Because of the proven insufficiency of mechanical treatment in peri-implantitis, adjunctive local antibacterial treatments have been explored. Chlorhexidine [37], despite being a potent antiseptic, showed disappointing results in a recent meta-analysis by Liu et al. [38]. They found no clear benefit of CHX in the nonsurgical management of peri-implant mucositis and could not draw conclusions in the nonsurgical management of peri-implantitis [36]. Adjunctive antimicrobials, such as minocycline and doxycycline, have outperformed CHX, with improvements in inflammatory changes and probing depths. However, long-term use of antimicrobials is not feasible, and novel approaches are welcome.

Strong evidence supports the use of aMMP-8 in oral fluids in the assessment of the activity and severity of peri-implant disease [39]. Peri-implant tissues consist principally of type I collagen, and MMP-8 is the predominant enzyme responsible for the degeneration of both peri-implant soft and hard tissues. An increased level of MMP-8 in oral fluids is associated with peri-implant disease, especially in a clinically active phase, but it must be emphasized that the levels of aMMP-8, but not latent or total MMP-8, reflect the peri-implant disease activity [38]. The aMMP-8 PoC test used in this study measures the active form of MMP-8 and has been widely validated as a numerical measurement of implant tissue status [24,25,26,27,28,29,30,31,32]. In this study, PISF collection was performed in a single sulcus site at the diseased implant, not circumferentially. The method of PISF collection can have an effect on the aMMP-8 results [40].

We observed a negative BOP in three studies at four weeks after dual-light aPDT use. The absence of BOP is a precise measurement for periodontal stability with a negative predictive value of 98% [41]. This is a clear indication of a reduction in inflammation in the tissues around the implant, and the simultaneous reduction in aMMP-8 levels strengthens the credibility of the observations. Most probably, the change reflects the observed reduction in the bacterial triggering load, measured by VPI. While the predictive value of BOP has limitations due to several reasons [42], we used a standardized tool for the BOP to ensure appropriate reliable measurement, including avoiding traumatic instead of pathologic induction of bleeding [42]. Because of the particularly low patient number in this early feasibility study, which resembles a case report series, a Gaussian distribution cannot be assumed and nonparametric statistical analysis methods were used.

Antibacterial photodynamic therapy and aBL have been presented as new antibacterial methods. Both methods provide a significant dose-dependent antibacterial action [10,16]. Nikinmaa et al. (2020) recently showed the advantage of combining aPDT and aBL [16]. This combination, called dual-light, showed a significantly increased antibacterial effect when compared with either part itself. Furthermore, they were able to produce a more sustained antibacterial effect of dual-light by inducing repeated use when compared with aPDT or aBL. Even though a lack of antibacterial resistance formation has been stated regarding aPDT and aBL [41], both methods show some adaptation when applied continuously [16]. The dual-light approach was shown to be significantly more resistant to bacterial adaptation [16].

The rapid development in LED technology has provided an opportunity to attack dental plaque [43] with light-based antibacterial treatment applications (www.lumoral.com; Koite Health LTD, Espoo, Finland). LED technology can bring the treatment location from clinics to home. Antibacterial photodynamic therapy is a potent antibacterial tool, but results in periodontitis have been generally disappointing. In most of these studies, the treatment has been applied only once [44]. In the few studies where the aPDT has been used repeatedly, only one or two repetitions have been applied at the early phase of the study period [45,46]. Treatment frequency can have a significant impact in results. The antibacterial action of aPDT, when compared head-to-head with scaling and root planing (SRP), has shown early results comparable with those of SRP [47]. We claim that the replacement of mechanical cleaning with aPDT is probably not an answer, but these results show the potential of its use as an adjunctive therapy. If this kind of efficacy can be brought even partly to home use, we can at least hope to see clinical benefits. The ability of the ICG in the mouth rinses to end up in the peri-implant pocket is probably somewhat limited [46], which can make a difference in in-clinic aPDT application. However, the antibacterial action, when measured as a plaque reduction in the supragingival plaque, seems to work. There is a limited number of studies in which several aPDT applications have been used [45]. In the studies where aPDT has been used several times, the results have been significantly better [46,47]. Indeed, a continuous application of aPDT can have benefits not shown in the current literature. To our knowledge, no significant side effects exist with aPDT, which subsequently could provide the best alternative to CHX treatment.

This is a small study with a limited population size and a relatively short study period of only one month. Our power calculations, based on a restricted number of available patient data, are weak and provide an initial reference at best. With an increasing number of patients being treated in small but cumulating pilot experiments, it is our aim to obtain further evidence for designing larger clinical trials with enough data to perform power calculations with better resolution and higher confidence. However, the observations reported here definitely encourage further studies. The use of a cross-over design would be a good option when studying a treatment such as Lumoral^®^ (Lumoral, Espoo, Finland), where the use of a placebo is particularly difficult [48]. Use of the dual-light aPDT method should not replace routine oral hygiene or standardized professional mechanical treatments but can act as an additional tool for preventing periodontal disease and supporting the treatment of already established disease. Dual-light therapy can be used by anyone, but it is most beneficial for people who need to improve the health of their connective tissues or for those for whom traditional tooth brushing is not possible or is challenging. There are several risk factors increasing implant failure, including higher age, smoking, diabetes or metabolic syndrome, previous head and neck radiation, and history of postmenopausal hormone replacement therapy [49,50,51]. Connective tissue infection progresses many times faster in implant teeth, and the healing process is more complicated than in natural teeth [52].

## 5. Conclusions

This pilot study suggests that dual-light therapy might be a promising adjunctive homecare tool to keep high-cost implants secure from peri-implantitis. It is easy and safe to use without any adverse effects. However, further studies are warranted to define the effectiveness of the treatment.

## Figures and Tables

**Table 1:** The patient characteristics.

Patient Characteristics (*n* = 7)	
Gender (M/F)	4/3
Age (years)	65−89
Smoking (yes/no)	0/7
Diabetes (yes/no)	0/7
Rheumatic (yes/no)	0/7
Asthma (yes/no)	1/6
Heart disease (yes/no)	2/5

## Data Availability

The data supporting the results reported here are available from the corresponding author upon request.
